# An Ensemble Feature Selection Approach-Based Machine Learning Classifiers for Prediction of COVID-19 Disease

**DOI:** 10.1155/2024/8188904

**Published:** 2024-04-17

**Authors:** Md. Jakir Hossen, Thirumalaimuthu Thirumalaiappan Ramanathan, Abdullah Al Mamun

**Affiliations:** ^1^Faculty of Engineering and Technology, Multimedia University, Melaka, Malaysia; ^2^Faculty of Information Science and Technology, Multimedia University, Melaka, Malaysia; ^3^School of Information and Communication, Griffith University, Nathan, Australia

**Keywords:** COVID-19 diagnosis, feature selection, machine learning

## Abstract

The respiratory disease of coronavirus disease 2019 (COVID-19) has wreaked havoc on the economy of every nation by infecting and killing millions of people. This deadly disease has taken a toll on the life of the entire human race, and an exact cure for it is still not developed. Thus, the control and cure of this disease mainly depend on restricting its transmission rate through early detection. The detection of coronavirus infection facilitates the isolation and exclusive care of infected patients. This research paper proposes a novel data mining system that combines the ensemble feature selection method and machine learning classifier for the effective identification of COVID-19 infection. Different feature selection approaches including chi-square test, recursive feature elimination (RFE), genetic algorithm (GA), particle swarm optimization (PSO), and random forest are evaluated for their effectiveness in enhancing the classification accuracy of the machine learning classifiers. The classifiers that are considered in this research work are decision tree, naïve Bayes, *K*-nearest neighbor (KNN), multilayer perceptron (MLP), and support vector machine (SVM). Two COVID-19 datasets were used for testing from which the best features supporting the dataset were extracted by the proposed system. The performance of the machine learning classifiers based on the ensemble feature selection methods is analyzed.

## 1. Introduction

A deadly respiratory illness known as coronavirus disease 2019 (COVID-19) caused by the SARS-CoV-2 coronavirus has lately gone global. The global outbreak of the COVID-19 pandemic has crippled the world economy and has brought about a devastating impact on the lives of the entire human race. Moreover, a specific and accurate cure for this deadly disease is still not discovered. Despite developing numerous vaccines, this infectious disease has not been completely eradicated. Most COVID-19 patients only experience mild to moderate symptoms, but 15% of them eventually develop severe pneumonia, and 5% go on to advance acute respiratory distress syndrome (ARDS), multiorgan failure, or septic shock [1]. Symptomatic management, oxygen therapy, and mechanical ventilation are the cornerstones of clinical treatment for individuals with respiratory arrest [2, 3]. The only way of controlling this disease is to decelerate its rapidly growing transmission rate. Decelerating the spread of COVID-19 infection depends on accurate, quick, inexpensive, and accessible detection of COVID-19 illness in an individual. This objective of deceleration is made possible through quick identification and isolation of the infected patients. Artificial intelligence-based software can be used to counter the increasing transmission rate of the deadly pandemic.

There has been research work done in applying machine learning algorithms for the classification of COVID-19 disease from sample datasets. Yan et al. [4] used various possible factors and demographic details to build an XGBoost model for predicting about the COVID-19 severity. In their work [4], the XGBoost showed an accuracy of 90%. Yao et al. [5] also used the support vector machine (SVM) classifier model to predict about the COVID-19 severity. In their work [5], the SVM showed an accuracy of 81.5%. Hu et al. [6] built a logistic regression model to predict about the COVID-19 severity. In their work [6], the logistic regression model showed an accuracy of 85%. The dataset used in the research works [4–6] is about the COVID-19 patients admitted at Tongji Hospital in 2020 [7]. Wong, Xiang, and So [8] also used the XGBoost classifier to predict about the COVID-19 severity from the dataset obtained from the United Kingdom Biobank (UKBB) [9]. In their work [8], the XGBoost showed an accuracy of 6.68%. Sun et al. [10] built a SVM model to predict about the COVID-19 severity. The data collected from the Shanghai Public Health Clinical Centre [11] were used for training the SVM classifier. In their work [10], the SVM showed an accuracy of 7.75%. An et al. [12] used different machine learning classifiers such as SVM, random forest, and *K*-nearest neighbor (KNN) classifiers for predicting about the COVID-19 severity. The data obtained from the Korean National Health Insurance Service [13, 14] were used for training the machine learning classifiers. In their work [12], the linear SVM model showed the best performance with an area under the receiver operating characteristic curve (AUC) of 96.2% when compared to the other classifiers. Zagrouba et al. [15] built a SVM model to predict about the COVID-19 severity. The dataset with 303 patients which was obtained from the World Health Organization was used for training the SVM classifier. In their work [15], the SVM model showed an accuracy of 96.7%. There also has been research work done on the identification of COVID-19 disease from medical images [16–19]. In [20], a computerized method for extracting crucial and reliable information about the diseased area scans to distinguish a healthy patient and a COVID-19-infected patient is proposed. Their method involves retraining a pretrained model with transfer learning to calculate characteristics from an average pooling and fully connected layers. Their method is utilized to fuse the most pertinent characteristics into one vector after which the classifier performs the final classification. In [21], a unique technique based on generating colored images is proposed from 12-lead paper-based ECG scans in 2D and feeding them into a modified CNN architecture to identify COVID-19, but their method results in more computational time. In [22], a powerful machine learning classifier that successfully discriminated COVID-19 CXR images from typical patients and viral pneumonia is established. x-rays are still the most common and quick screening method for lung infections and illnesses among all imaging modalities. However, some suspected lung infection lumps can be shown in x-ray scans, which could lead to a false positive. In [23], E-DiCoNet is a different model that diagnoses COVID-19 without the need for a sizable dataset, because the technique collects spatial data from instances and object fragments utilizing probable changes in the objects' existence. Some of the research challenges in analyzing the COVID-19 datasets are to investigate the effectiveness of the ensemble feature selection method in the classification of COVID-19 disease and to analyze the performance of various feature selection methods and machine learning classifiers by using large COVID-19 datasets.

In this paper, an ensemble feature selection-based machine learning classification (EFS-MLC) system is proposed for the classification of COVID-19 disease. In the proposed system, the traditional machine learning classifiers such as decision tree, naïve Bayes, KNN, multilayer perceptron (MLP), and SVM are used in the classification of COVID-19 disease from the sample datasets. The ensemble feature selection method used with the proposed system is based on different feature selection techniques such as the chi-square test, recursive feature elimination (RFE), genetic algorithm (GA), particle swarm optimization (PSO), and random forest are used to identify the best features from the datasets. Classification is the most prominent challenge in machine learning-based techniques, which is utilized in determining to which class the obtained observations belong. Decision tree [24] is a nonparametric technique for regression and classification analysis which uses both continuous and categorical output. The regression tree model is used to deal with continuous data. A probabilistic classifier called naïve Bayes classifier [25] uses a straightforward effective machine learning approach. Naïve Bayes has performed admirably in a number of challenging real-world applications. Similarly, KNN [26] is employed to train the dataset and classify it using similarity and distance metrics. KNN points with numerous nearest neighbor and distance metrics. MLP [27] is engaged due to its advantages like learning capability and accurate classification towards datasets. In consequence, SVM [28] is a classifier which results in providing excellent accuracy rate towards unbalanced data.

The rise in the number of variables employed during sophisticated data analysis is one of the key issues that occur. Too many variables in an analysis frequently necessitate a vast memory space and speed. The goal of feature extraction is to use fewer resources to describe massive datasets. In feature selection techniques, the features are extracted from the processed output and are engaged with a feature extraction process utilizing different techniques to obtain robust and improved features owing to the small quality of data to be trained. The feature extraction process is used to select the minimum number of features which guarantees the improved level of accuracy. The feature extraction results in reducing the generalizability mistake while obtaining a more extensively tested experiment. On engaging the chi-squared technique [29], low computation time is achieved with the flexibility to handle more data along with robustness in the distribution of data. The RFE technique [30] aids in the identification of factors determining the kernels based on weights of radial function. Utilization of a GA results in providing significantly effective features in contrast to another search engine over a large search [31]. The optimization strategies are introduced to extract COVID-19 features, which include PSO [32] having few parameters to tune the classifier and attain the best practical solutions. The random forest technique [33] supports in accurate extraction of features by using a number of trained decision trees.

The major focus of this research paper is to present an effective disease prediction system to facilitate the accurate classification of COVID-19 infections. Here, the performance of several machine learning-based classifiers in classifying COVID-19 disease is analyzed. Additionally, several feature selection techniques are also examined for their effectiveness in improving the classifier performance.

## 2. The Proposed System

**Figure 1:**
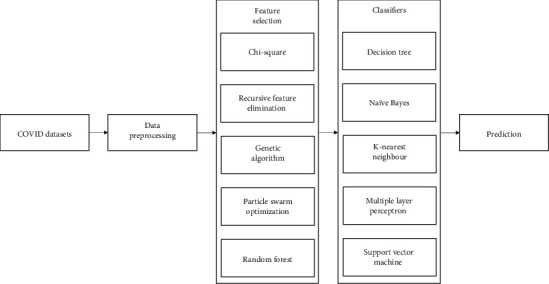
Architecture of the proposed system.

Figure 1 shows the architecture of the EFS-MLC system. According to the EFS-MLC system design, initially, the datasets containing the samples about COVID-19 patients are given as input to the detection model. The input dataset comprises numerous missing data, which are in turn predicted and substituted with the aid of preprocessing. After preprocessing the dataset, the process of feature selection is accomplished using different techniques including chi-square, RFE, GA, PSO, and random forest. The selected optimal features are used for training machine learning classifiers such as decision tree, naïve Bayes, KNN, MLP, and SVM.

### 2.1. Feature Selection

Feature selection is the technique in which the relevant attributes that support the precise detection of COVID-19 infections are obtained. In some cases, the process of feature selection is crucial owing to its role in improving classification accuracy. The ensemble feature selection approach is used in the EFS-MLC system where the best features are selected through a majority voting method by using chi-square, RFE, GA, PSO, and random forest methods. The feature selection methods considered in the EFS-MLC system are described below.

1. *Chi-squared test.* The chi-squared test is determined on the basis of the association between two variables. Moreover, this technique is mainly preferred for datasets comprising categorical features. It estimates the chi-square score of every feature by evaluating the degree of association between the target and each variable. Then, the features with the best chi-square score are selected. The chi-square score is expressed as shown in Equation (1).
  (1) $$\begin{array}{c} X^{2}=\frac{{\left(O-E\right)}^{2}}{E} \end{array}$$

Here, the observed frequency (*O*) is the number of experimental data, and the expected frequency (*E*) is the probability count of each data. The chi-squared process is carried out by specifying the hypothesis initially. Then, it is followed by devising an analysis plan, and finally, the result is deduced after examining the sample data.

2. *RFE.* In the RFE method, the features are prioritized by ranking them on the basis of their estimated importance. Thereby, only the most relevant features are sustained, and the least relevant features are eliminated. It is first addressed how to choose features for linear binary classifiers. A linear classifier has the form of an unknown input vector *x*, as shown in Equation (2). Here, *w* and *b* are the weight vector and bias, respectively.
  (2) $$\begin{array}{c} y\left(x\right)=\mathrm{sign}\left(w.x-b\right) \end{array}$$

Evidently, the most informative features are associated with the input items that are weighted by the highest absolute value. The least weighted inputs can therefore be eliminated with little effect on the classification outcome if the classifier has been properly trained. This concept is carried out by feature ranking in feature selection.

3. *GA.* The natural selection and genetics theories underlie how the GA search algorithm operates. This search algorithm is frequently employed in the feature selection process to obtain optimal features through subset evaluation. The benefit of utilizing GA is that, in contrast to other search algorithms, it conducts a global search rather than using greedy and local search methods. As a result, GA is a useful method for solving feature selection issues because it yields high-quality results. To create a new population, it uses the crossover, mutation probability in addition to survival of the fittest procedures until the highest criterion is reached. Chromosomes are employed to build a population when the GA is applied, and these chromosomes are used to represent the feature. Because it is used to assess each person's robustness, fitness value is a crucial component of GA. It is possible to determine the fitness value in this investigation using Equation (3).
  (3) $$\begin{array}{c} \text{Fitness}=W_{A.\text{accuracy}}+W_{nb/N} \end{array}$$

Here, *W*_*nb*_ the weight of *N* features is considered in the classification, and *W*_*A*_ is the weight of accuracy, with  *N* ≠ 0. After determining the fitness function for each chromosome, crossover and mutation are used to affect the population. In contrast to mutation, which involves creating new persons through gene random selection from a chromosome, crossover involves the random selection of two-parent genes to create genes depending on fitness function score. The fittest people are those with the lowest fitness values.

4. *PSO.* A heuristic search or optimization technique called PSO is influenced by the cooperative behaviour of bird or fish schools. Birds or fish, here referred to as particles, interact with one another through a communication system that is incredibly complex. The particles are distinguished by a sort of updating their best prior performances in the present flight, in addition to their communication abilities. A predetermined number of particles are involved in a typical PSO setup. Each particle is connected to a vector that has a set number of elements. These components are initially initialized, and then the entire particle system undergoes iterative processing. By replacing the values of the vector's elements for those in the goal function, the performance of each particle is assessed at the conclusion of each iteration in terms of how closely it adheres to the objective function. Occasionally, one of the particles emerges with the greatest outcomes at the end of each iteration. This particle is referred to as the iteration's top particle overall. Another particle might overtake the current top particle in the subsequent iteration. Additionally, each particle may perform differently over the course of several iterations, and by taking into account both the previous and the current iteration, a certain combination of the vector's elements may stand out as the vector for that particular particle that is performing the best overall. The said particle's personal best is this specific vector. Every particle is updated with the best vector available to produce the greatest outcomes.

Thus, following the update procedure that concludes each iteration, each particle will assume its best vector to date, corresponding to its personal best and one becomes the best particle overall. A velocity is computed and added up with the updated best-performing vector of each particle based on the vector of the individual best performance of each particle and the vector of the particle with the best overall performance. As a result, at the conclusion of each cycle, all particle vectors are updated, and performance is assessed using Equations (4) and (5).

(4) $$\begin{array}{c} v_{d}^{i}=w\left(G\right)\times v_{d}^{i}+c_{1}\times r\text{and}1_{i}^{d}\times \left({p\text{best}}_{i}^{d}-X_{i}^{d}\right)+c_{2}\times \left({g\text{best}}_{i}^{d}-X_{i}^{d}\right) \end{array}$$

(5) $$\begin{array}{c} X_{i}^{d}=X_{i}^{d}+V_{i}^{d} \end{array}$$

The components of each particle's vector are gradually altered as the iterative process is carried out, and they all move in separate ways towards the shared objective before coming together at a specific location. At this particular point, all of the particles' vector elements are going to be the same, and their performances relative to the objective function will be practically identical. The necessary solution is that the elements of the vector for every particle will be the same at the point of convergence. The best feature is selected initially by assigning primary values, along with the estimation of fitness value for every particle, and the current fitness values are achieved. If the attained value is better than the fitness value achieved before, it is updated as the current value. In case the previous value is better, the algorithm terminates. The process is repeated until the optimal solution is achieved.

5. *Random forest.* The random forests approach employs a group of decision tree classifiers and it uses bootstrap samples for training each tree. From a random attribute subset, the selection of each split attribute is accomplished. Individual classification is based on the total votes cast by all of the forest's trees. The construction of each tree is achieved using *M* explanatory attributes and *N* people data:
  • Select *N* people with replacement as your training sample from the full dataset.
  • Pick *m* attributes at random from all of the data's *M* attributes and place them at each node of the tree. The dataset's total number of characteristics determines the absolute magnitude of *m*, which depends on *M* and stays constant over the course of forest construction.
  • Pick the *m* attributes subset from the above list that best fits the split at the current node.
  • Repeat the above two steps till the tree reaches its full growth (no pruning).

The model becomes more random as trees grow, owing to random forests. When splitting a node, it finds the best feature from a random subset of features, not the most significant one. As a result, a model with a broad range of diversity tends to be better. The random forest classifier selects the most important features based on a given entropy value, and the model is rebuilt with the selected features.

### 2.2. Classifiers

In the EFS-MLC system, several machine learning classifiers are employed for the prediction of COVID-19 infections which are described below.

1. *Decision tree.* A decision tree is a tree-like structure consisting of root nodes, internal nodes, and leaf nodes which are applied for modelling decisions and their outcomes. Attribute tests are represented by internal nodes of the decision tree, the results are represented by branch nodes, and the class labels are represented by leaf nodes. Decision tree is useful in many situations because development does not require domain-specific expertise. The decision tree classifier is unique not only in efficiency and speed but also in its design and modification. As a result, the decision tree has higher accuracy compared to the unit classification principle. Choosing an appropriate tree size is the most important step in adopting the decision tree approach. There are two basic problems when using decision trees in data mining. Large trees cause overfitting, and small trees cause underfitting. When a decision tree is truncated, information is less important than test data. Removing data during processing improves the accuracy of results and reduces data size. Thus, the pruning concept effectively removes the classification approach complexity. There are various measures such as entropy, Gini index, and classification error which are used for splitting the internal nodes. These measures depend on the degree of impurity of the child nodes. The decision tree used in the EFS-MLC system uses the Gini index as the splitting criterion with a maximum split of 100. The Gini index is given in Equation (6). Here, *p*(*i*/*t*) represents the fraction of records belonging to class label “*i*” at a given node “*t*” and *c* represents the number of classes.

(6) $$\begin{array}{c} \text{Gini}\left(t\right)=1-\overset{c-1}{\underset{0}{\sum }}{\left[p\left(i/t\right)\right]}^{2} \end{array}$$

2. *Naïve Bayes.* The naïve Bayes algorithm which is based on the Bayes' theorem extracts patterns from the training dataset with an assumption that all the input attributes of the dataset are conditionally independent. The naïve Bayes classifier classifies each of the test sample by computing the posterior probability, *P*(*X*|*Y*) as stated in Equation (7). Here, *P*(*X*|*Y*) represents the class conditional probability, *X* represents the input attribute set, *Y* represent the class, *d* represents the number of input attributes, and *y* represents the class label. The conditionally independence assumption for computing *P*(*X*|*Y*) is stated in Equation (8).

(7) $$\begin{array}{c} P\left(Y\left|X\right.\right)=\frac{P\left(Y\right)\prod_{i=1}^{d}P\left(X\left|Y\right.\right)}{P\left(X\right)} \end{array}$$

(8) $$\begin{array}{c} P\left(X\left|Y\right.=y\right)=\overset{d}{\underset{i=1}{\prod }}P\left(X_{i}\left|Y\right.=y\right) \end{array}$$

The Gaussian distribution is used more often to deal with class conditional probability for continuous attributes. The class conditional probability is stated in Equation (9) for Gaussian distribution. Here, *μ* represents mean, and *σ*^2^ represents variance.

(9) $$\begin{array}{c} P\left(X\left|Y\right.\right)=\frac{1}{\sqrt{2\pi \sigma }}\exp^{-{\left(x-\mu \right)}^{2}/2\sigma^{2}} \end{array}$$

3. *KNN.* The KNN algorithm is based on the neighboring data values that are given from the training dataset. The number of nearest neighbor data values which are needed to be considered is given as a parameter value when using the KNN classifier. The KNN algorithm determines how to classify a given test sample based on the number of “*K*” values or nearest neighbors. KNN models classify the training dataset directly. This means new instances are predicted by finding the class labels of the majority of “*K*” neighboring data from the training set. The process of assigning the class label to a new test sample based on the majority class labels of the neighboring data is stated in Equation (10). Here, *y*′ represents the class label of the test sample, *v* represents the majority class labels of the neighboring data, *y*_*i*_ represents the class label of nearest neighbor *x*_*i*_, and *I*(.) is an indicator function. KNN classifier with three neighboring data values is used in the EFS-MLC system.

(10) $$\begin{array}{c} y^{\text{'}}=\arg\underset{v}{\max}\underset{\left(x_{i},y_{i}\right)\in D}{\sum }I\left(v=y_{i}\right) \end{array}$$

4. *MLP.* MLP is a neural network with one or more layers of hidden neurons. There are three layers: an input layer (supplied with data variables), a hidden layer (containing data manipulation functions), and an output layer (holding predicted values). Each neuron in the hidden layer and output layer computes the weighted sum of the input signal and compares it with the threshold value *θ* using the sign activation function as stated in Equation (11). Here, *x*_*i*_ is the value of input *i*, *w*_*i*_ is the weight value of input *i*, *n* is the number of neuron inputs, and *Y* is the output of neuron *i*.

(11) $$\begin{array}{c} Y=\mathrm{sign}\left[\overset{n}{\underset{i=1}{\sum }}x_{i}w_{i}-\theta \right] \end{array}$$

There are different types of activation functions such as step, sign, linear, and sigmoid. The backpropagation learning algorithm is most commonly used in MLP. In the backpropagation algorithm, initially, the weights are initialized for each input. Then, the input pattern is propagated from layer to layer until the output layer is produced from the output layer. Then, the error is calculated by comparing the output pattern with the actual output. Based on the error, the patterns are propagated backward from the output layer to the input layer, and weights are modified. This process continues until the error value is minimized. The MLP is used in the EFS-MLC system with one hidden layer consisting of five neurons that use rectified linear units [34] activation function.

5. *SVM.* SVM uses boundary values to generate a hyperplane in multidimensional space for each class label. SVM aims to maximize class breaks by optimally separating hyperplanes. The hyperplane is used as the data instance for the support vectors of the given dataset. An edge is defined as the shortest distance between a support vector and a hyperplane. Linear SVMs can be used for classification in scenarios where a given dataset is linearly constrained. If your dataset is nonlinearly constrained, you can use nonlinear SVM. The SVM classifier classifies the test sample as shown in Equation (12). Here, *y* is the class label of the test sample, *w* and *b* are the parameters of the SVM model, and *z* is the test sample. The SVM classifier with linear kernel function is used in the EFS-MLC system

(12) $$\begin{array}{c} y=\left\{\begin{array}{cc} +1 & \text{if }w∙z+b>0\\ -1 & \text{if }w∙z+b<0 \end{array}\right. \end{array}$$

## 3. Results and Discussion

The EFS-MLC system is tested with two COVID-19 datasets. One dataset is retrieved from the Israeli Ministry of Health website [35]. The other dataset called the symptoms and COVID-19 presence dataset (May 2020 data) is retrieved from the Kaggle website [36]. The Israeli COVID-19 dataset consists of 101,796 samples. The input attributes of the Israeli COVID-19 dataset are cough, fever, sore throat, shortness of breath, headache, age of 60 years and above, gender, and test indication. The target variable of the Israeli COVID-19 dataset is a positive or negative result for COVID-19 disease. The symptoms and COVID-19 presence dataset consist of 5434 samples. The input attributes of symptoms and COVID-19 presence dataset are breathing problem, fever, dry cough, sore throat, running nose, asthma, chronic lung disease, headache, heart disease, diabetes, hypertension, fatigue, gastrointestinal, abroad travel, contact with COVID-19 patient, attended large gathering, visited public exposed places, family working in public, exposed places, wearing masks, and sanitation from market. The target variable of symptoms and COVID-19 presence dataset is whether the COVID-19 disease is present or not. The input attributes in both the COVID-19 datasets are of nominal data type.

The missing values found in the Israeli COVID-19 dataset are addressed by using the *K*-mean imputing technique [37]. The Israeli COVID-19 dataset is also an imbalanced dataset. It is converted into a balanced dataset by using the *K*-mean SMOTE technique [38].  Table 1 shows the features selected by each of the feature selection method used in the EFS-MLC system. A maximum of five best features were extracted from the Israeli COVID-19 dataset, and a maximum of seven best features were extracted from the symptoms and COVID-19 presence dataset. The ensemble feature selection method works by selecting the best features that get majority votes of the 5 different feature selection methods employed in the EFS-MLC system. For the Israeli COVID-19 dataset, the best features selected by the ensemble feature selection method are cough, fever, sore throat, headache, and test indication. For the symptoms and COVID-19 presence dataset, the best features selected by the ensemble feature selection method are breathing problem, fever, dry cough, sore throat, abroad travel, contact with COVID-19 patient, and attended large gathering.

The datasets are split into training and testing datasets in an 80:20 ratio, respectively. The training dataset is used to train the classifier. The trained classifier is tested using the testing dataset. In the EFS-MLC system, the classification accuracy is used as the fitness function for the GA-based feature selection method and the ensemble classifier is used as an estimator for the RFE feature selection method and PSO-based feature selection method. The performance of the classifiers is analyzed by using various measures such as classification accuracy, precision, recall, *f*1-score, and AUC [39].  Table 2 shows the performance of different machine learning classifiers for the Israeli COVID-19 dataset before using the feature selection methods.  Table 3 shows the performance of different machine learning classifiers for the feature subset of two COVID-19 datasets generated by the ensemble feature selection method. The machine learning classifiers show poor recall and *f*1-scores for the Israeli COVID-19 dataset as shown in Table 2. The recall and *f*1-scores of machine learning classifiers are improved for the Israeli COVID-19 dataset after converting the imbalanced Israeli COVID-19 dataset into a balanced dataset and after using the ensemble feature selection method as shown in Table 3. The naïve Bayes shows an average performance when compared to other machine learning classifiers for the symptoms and COVID-19 presence dataset even after using the feature selection method as shown in Table 3. It can be seen from Tables 2 and 3 that the classification accuracy for the Israeli COVID-19 dataset is improved and the classification accuracy for the symptoms and COVID-19 presence dataset is slightly reduced after using the ensemble feature selection method. The best features that contribute towards the two COVID-19 datasets are extracted using the ensemble feature selection method based on chi-square, RFE, GA, PSO, and random forest in the proposed EFS-MLC system.

**Figure 2:**
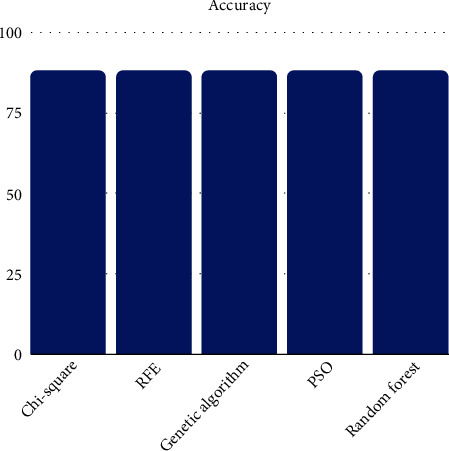
Performance comparison of feature selection methods for the Israeli COVID-19 dataset.

**Figure 3:**
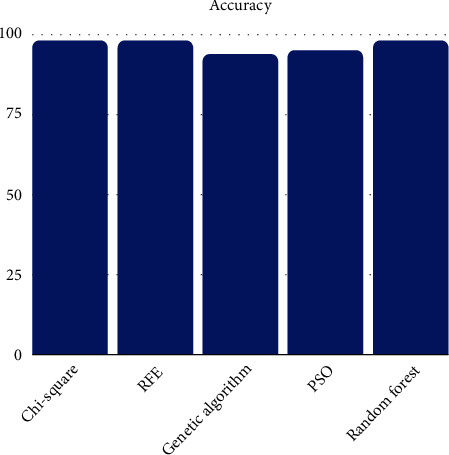
Performance comparison of feature selection methods for symptoms and COVID-19 presence dataset.

Figures 2 and 3 compare the accuracy of different feature selection methods when using an ensemble machine learning classifier for the selected feature subset of the Israeli COVID-19 dataset and symptoms and COVID-19 presence dataset, respectively. The ensemble machine learning classifier classifies the data through a voting method based on different employed classifiers such as decision tree, naïve Bayes, KNN, MLP, and SVM. The chi-square, RFE, GA, PSO, and random forest show the same level of accuracy for the Israeli COVID-19 dataset as shown in Figure 2. The chi-square, RFE, and random forest feature selection methods show slightly improved accuracy when compared to the GA and PSO feature selection methods for the symptoms and COVID-19 presence dataset as shown in Figure 3. The performance of the classifiers differs when testing with the two COVID-19 datasets. The trained classifiers show better performance for the symptoms and COVID-19 presence dataset when compared to the Israeli COVID-19 dataset. This difference is because of the dataset size. The Israeli COVID-19 dataset has a larger number of samples when compared to the symptoms and COVID-19 presence dataset.

## 4. Conclusion

A crucial component of better pandemic management is the early identification, isolation, and care of people. The EFS-MLC system proposed in this paper supports for identifying the most promising combination of feature selection and classification approach suitable for developing an effective prediction system that is accurate in detecting COVID-19 infections. Several feature selection techniques like chi-square, RFE, GA, PSO, and random forest were applied in an ensemble-based approach for identifying the most important features in the COVID-19 datasets and enhancing the operation of machine learning classifiers which includes decision tree, naïve Bayes, KNN, MLP, and SVM. The KNN classifier based on the ensemble feature selection approach showed a little improved performance when compared to other classifiers for the Israeli COVID-19 dataset. The employed machine learning classifiers showed a similar classification accuracy of 88.8% for the symptoms and COVID-19 presence dataset when using the ensemble feature selection approach. Also, the ensemble feature selection approach used in the proposed EFS-MLC system has extracted the best features of the Israeli COVID-19 dataset and symptoms and COVID-19 presence dataset which is evident through the performance of different machine learning classifiers employed in the proposed system.

## Figures and Tables

**Table 1:** Features extracted using different feature selection methods.

**Feature selection methods**	**Israeli COVID-19 dataset**	**Symptoms and COVID-19 presence dataset**
Chi-square	1. Cough 2. Fever 3. Sore throat 4. Headache 5. Test indication	1. Breathing problem 2. Fever 3. Dry cough 4. Sore throat 5. Abroad travel 6. Contact with COVID-19 patient 7. Attended large gathering

RFE	1. Cough 2. Sore throat 3. Headache 4. Age of 60 years and above 5. Test indication	1. Breathing problem 2. Fever 3. Dry cough 4. Sore throat 5. Abroad travel 6. Contact with COVID-19 patient 7. Attended large gathering

GA	1. Cough 2. Sore throat 3. Headache	1. Breathing problem 2. Sore throat 3. Asthma 4. Chronic lung disease 5. Abroad travel 6. Contact with COVID-19 patient 7. Wearing masks

PSO	1. Cough 2. Fever 3. Sore throat 4. Shortness of breath 5. Gender	1. Breathing problem 2. Fever 3. Dry cough 4. Sore throat 5. Running nose 6. Abroad travel 7. Visited public exposed places

Random forest	1. Cough 2. Fever 3. Sore throat 4. Headache 5. Test indication	1. Breathing problem 2. Fever 3. Dry cough 4. Sore throat 5. Abroad travel 6. Contact with COVID-19 patient 7. Attended large gathering

**Table 2:** Performance of classifiers before feature selection.

**Datasets**	**Classifiers**	**Accuracy**	**Precision**	**Recall**	**f**1**-score**	**AUC**
Israeli COVID-19 dataset	Decision tree	0.83	0.95	0.23	0.37	0.78
	Naïve Bayes	0.83	0.95	0.22	0.36	0.71
	KNN	0.83	0.95	0.22	0.36	0.74
	MLP	0.83	0.95	0.23	0.37	0.78
	SVM	0.83	0.95	0.23	0.37	0.78

Symptoms and COVID-19 presence dataset	Decision tree	0.99	0.99	0.99	0.99	1.0
	Naïve Bayes	0.78	1.0	0.73	0.84	0.99
	KNN	0.99	1.0	0.99	0.99	1.0
	MLP	0.98	0.98	0.99	0.99	1.0
	SVM	0.99	0.99	0.99	0.99	0.99

**Table 3:** Performance of classifiers after feature selection.

**Datasets**	**Classifiers**	**Accuracy**	**Precision**	**Recall**	**f**1**-score**	**AUC**
Israeli COVID-19 dataset	Decision tree	0.88	1.0	0.77	0.87	0.93
	Naïve Bayes	0.88	1.0	0.77	0.87	0.9
	KNN	0.88	1.0	0.77	0.87	0.88
	MLP	0.88	1.0	0.77	0.87	0.93
	SVM	0.88	1.0	0.77	0.87	0.9

Symptoms and COVID-19 presence dataset	Decision tree	0.97	0.97	1.0	0.98	1.0
	Naïve Bayes	0.78	1.0	0.72	0.84	0.99
	KNN	0.98	0.98	0.99	0.99	0.98
	MLP	0.97	0.97	1.0	0.98	1.0
	SVM	0.97	0.97	1.0	0.98	0.99

## Data Availability

Two COVID-19 datasets were used in this study which were retrieved from the Israeli Ministry of Health and the Kaggle websites. The dataset which is retrieved from the Israeli Ministry of Health website can be accessed using the following link: https://data.gov.il/dataset/covid-19/resource/74216e15-f740-4709-adb7-a6fb0955a048. The dataset which is retrieved from the Kaggle website can be accessed using the following link: https://www.kaggle.com/datasets/hemanthhari/symptoms-and-covid-presence.
